# The rise and fall of dental therapy in Canada: a policy analysis and assessment of equity of access to oral health care for Inuit and First Nations communities

**DOI:** 10.1186/s12939-017-0631-x

**Published:** 2017-07-20

**Authors:** Victoria Leck, Glen E. Randall

**Affiliations:** Health Policy and Management, DeGroote School of Business, McMaster University, 1280 Main Street West, Hamilton, ON L8S 4M4 Canada

**Keywords:** Dental health, Oral health, Health policy, Inuit, First nations, Dental therapy

## Abstract

**Background:**

Inequality between most Canadians and those from Inuit and First Nations communities, in terms of both access to oral health care services and related health outcomes, has been a long-standing problem. Efforts to close this equity gap led to the creation of dental therapy training programs. These programs were designed to produce graduates who would provide services in rural and northern communities. The closure of the last dental therapy program in late 2011 has ended the supply of dental therapists and governments do not appear to have any alternative solutions to the growing gap in access to oral health care services between most Canadians and those from Inuit and First Nations communities.

**Methods:**

A policy analysis of the rise and fall of the dental therapy profession in Canada was conducted using historical and policy documents. The analysis is framed within Kingdon’s agenda-setting framework and considers why dental therapy was originally pursued as an option to ensure equitable access to oral health care for Inuit and First Nations communities and why this policy has now been abandoned with the closure of Canada’s last dental therapy training school.

**Results:**

The closure of the last dental therapy program in Canada has the potential to further reduce access to dental care in some Inuit and First Nations communities. Overlaps between federal and provincial jurisdiction have contributed to the absence of a coordinated policy approach to address the equity gap in access to dental care which will exacerbate the inequalities in comparison to the general population. The analysis suggests that while a technically feasible policy solution is available there continues to be no politically acceptable solution and thus it remains unlikely that a window of opportunity for policy change will open any time soon.

**Conclusion:**

In the absence of federal government leadership, the most viable option forward may be incremental policy change. Provincial governments could expand the scope of practice for dental hygienists in the hope that it may support enhanced access, consumer choice, and efficiency in the delivery of oral health care to Inuit and First Nations communities in Canada.

## Background

While health care in Canada is generally of high quality, there are several vulnerable subpopulations that continue to experience poor health compared to the average Canadian. Of particular interest are the extreme health inequities experienced by Inuit and First Nations people, which include higher rates of diabetes, smoking behaviours, oral diseases, respiratory diseases, higher infant mortality rates, and lower life expectancy [1]. One particular equity concern is access to dental services. Oral health care is of particular importance because it is positively correlated with general health status [2]. Children specifically may be among the most vulnerable in terms of the negative impact poor oral health has on their overall health and development [3]. In some cases, poor oral health is so common among Inuit and First Nations children that researchers have found that caregivers may consider the high prevalence of dental decay among youth to be ‘normal’ and accept limited access to care and resulting poor outcomes without question [4].

In the late 1960s, the Canadian federal government convened a panel to examine how best to meet the oral health needs of Canadians. From this initiative, it was noted that there was a lack of dental providers in rural and remote areas, particularly in Inuit and First Nations communities, and this concern was identified as a priority issue to be addressed. The resulting Government of Canada report [5] recommended the establishment of a new type oral health care provider in Canada: the dental therapist. Dental therapists had already been used in other countries to address access-to-care issues and the Canadian committee felt that the ability of this type of provider to deliver core oral health services such as extractions, fillings, and preventive care would help to address the gap in access to services, particularly in Canada’s Inuit and First Nations communities. In the years that followed, the federal government funded two programs where small numbers of dental therapists were trained with the specific goal of having these individuals providing services in rural and remote locations. However, as government priorities shifted, less attention was given to dental therapists as a policy approach for dealing with oral health access and equity issues. In November 2011, the last dental therapy program in Canada was closed. The termination of this training program raises concerns about how vulnerable Indigenous people in remote communities will obtain access to adequate oral health services, and how we might prevent a widening of the already enormous oral health disparity between Indigenous and non-Indigenous people.

This paper explores the rise and fall of the dental therapy profession in Canada and the resulting impact on Inuit and First Nations communities in terms of access to basic oral health care. It begins by providing background information that places the importance of oral health, and the lack of equitable access to services for Inuit and First Nations communities, into context. A discussion of the state of policy analysis with respect to equity issues is provided along with a description of the methods and agenda-setting framework that guided the analysis. A policy analysis, that elaborates on the initial use of dental therapy as a policy solution to equity concerns, is conducted using a range of historical and policy documents. In particular, the paper explores why dental therapy was originally pursued as a policy option to ensure equitable access to oral health care for Indigenous communities and why this policy has now been abandoned with the closure of Canada’s last dental therapy training school. The paper concludes with a possible policy option moving forward that may support enhanced equity of access, consumer choice, and efficiency in the delivery of oral health care to Inuit and First Nations communities in Canada and insights on the likelihood of such a policy option being implemented.

## Methods

There is an extensive body of research addressing a wide range of equity issues, including those relating to Inuit and First Nations communities in Canada. However, there is limited work that assesses these issues from a policy lens using health policy analysis theories and frameworks to build a more nuanced understanding of the policy process and to explain policy outcomes [6]. In general, health policy analysis is a multi-disciplinary approach that helps to explain the policy process and outcomes by analyzing the interactions among ideas, interests and institution. It has been argued that, in the absence of appropriate health policy analysis, achieving health reforms will be extremely difficult [6–8]. Some policy analysis approaches have focused on the role of “path dependence” [9], or “advocacy coalitions” [10], while others have used the notion of “punctuated-equilibrium” [11] to explain policy processes and outcomes. For many policy analysts the first and most crucial step towards understanding policy formation is understanding how and when an issue makes its way to the government’s decision agenda.

The policy analysis framework used here is John Kingdon’s agenda-setting theory [12]. This theory argues that multiple events (or streams) must align for a “window of opportunity” to open up for an issue to reach the government’s decision agenda. These are the “*problem*”, “*policy*”, and “*politics*” streams. The *problem* stream identifies when an issue has become of such public importance that government feels compelled to take action. The *policy* stream consists of one or more technically acceptable policy option being feasibly available should action be taken. The *politics* stream encompasses the political acceptability of taking action which may be influenced by the national mood or a change in governing political party. In the absence of alignment of these streams policy change will be incremental or non-existent.

Given that the policy problem and solution that is being analyzed unfolds over an extended period of time, we blend an historical policy analysis with Kingdon’s framework. The historical analysis adopts the critical approach which “views all social phenomena and historical events from the point of view of continually changing systems of social relationships and dependencies” [13]. This approach supports an analysis that takes into consideration the broader context within which a policy exists.

An electronic search was conducted to identify web sites and documents. Key words used in the search were “dental therapy” and “dental therapists”. Web sites, documents, and government debates were reviewed to identify material that pertained to dental therapy in Canada. While there was a dearth of academic literature on the topic as it pertained to the Canadian context, numerous relevant documents and reports were identified through association and school web sites. These documents were reviewed to establish time-lines, key participants, and the extent to which they contributed to our understanding of the problem, policy and politics streams.

### The problem stream: Oral health and equitable access to care for inuit and first nations communities

Good oral health is extremely important because it is closely associated with one’s overall health. For instance, dental decay and gum disease have been linked to diabetes and respiratory diseases, which are particularly prevalent among Inuit and First Nations populations [1]. Studies have also identified a positive correlation between poor oral health and heart disease [14] as well as between poor maternal oral health and the likelihood of delivering pre-term, low-birth-weight babies [3, 6]. Alongside these general health concerns, the negative societal effects of poor oral health are problematic. According to the *Canadian Health Measures Survey* (CHMS), pain and infection associated with oral disease can hinder one’s ability to attend school or gain employment, negatively affecting important social and economic areas of life [15].

Inuit and First Nations peoples in Canada tend to experience a greater burden of disease compared to their non-Indigenous counterparts. The 2008–2009 *Inuit Oral Health Survey* (IOHS), which included areas of Canada’s Arctic (excluding Nunavik), revealed that, among the 1216 Inuit people surveyed, ranging from three to forty plus years of age, the preventable chronic disease of tooth decay was two to three times worse than that of the average Canadian [16]. Compared to Canadians living south of the sixtieth parallel, the IOHS reported poorer oral health and higher frequency of food avoidance due to oral pain [15]. Similarly, the *First Nations Oral Health Survey* (FNOHS) reports that individuals residing on reserves in eight communities across Canada have poorer oral health compared to the non-Indigenous population [17].

In Canada, oral health care is primarily paid for by third-party insurers or by the clients themselves through out-of-pocket payment and is not covered by provincial universal health care insurance plans. In 2011, more than $11 billion was spent on oral health care in Canada. According to *National Health Expenditure Trends*, from 1975 to 2011, 59.2% of oral health care was paid through insurance (both private plans and public programs), with the remaining 40.2% coming directly from clients [18]. However, Inuit and First Nations people in Canada typically receive health care benefits through the Non-Insured Health Benefits Program (NIHB), a federal government program that provides coverage for drugs, oral health care, vision care, medical supplies, and equipment to Indigenous people who are not covered under provincial health insurance plans [19]. Funding of these health care services removes the burden of out-of-pocket payment from the beneficiaries of the program, but the program is costly for the federal government. In 2011–2012, total NIHB expenditures were in excess of $1 billion. This included more than $333 million (31%) in medical transportation costs [19]. Oral health care was the third-largest expenditure out of all NIHB health care services, at a cost of $219.1 million (20.4%). Nunavut reported the highest rate of oral day surgery for children, at 97.2 per 1000 people, followed by Northwest Territories at 51.8. In comparison, Ontario had a rate of 8.4 [20].

The IOHS and the FNOHS reported that financial considerations were not the main reason for avoiding a visit to an oral health professional. The FNOHS (2011) found that “just 2.0% of children and 5.8% of adolescents and adults said they avoided going to a dental professional because of the costs involved, and 2.1% of children and 5.4% of adolescents and adults said they declined recommended care because of the cost” [[17], p. 18]. In addition, participants had “poor access to dental treatment and prevention services, especially in more remote communities” [[17], p. 4]. The IOHS (2011) reported that fewer than half of participants scheduled an appointment for oral health care even though very few identified costs as a factor in avoiding visiting or accepting recommended treatment. Cost also seems to be less of a factor in seeking oral health care because of Indigenous people’s coverage under the federal NIHB program [16].

The main obstacle to appropriate care appears to be access to appropriate health care professionals. As is the case with many different health care providers in Canada, there is an inequitable distribution of oral health human resources in northern Canada, with population demand exceeding the supply of local oral health care professionals. Many communities in northern Canada do not have a dentist residing in the community at all. For instance, in Nunavut, itinerant dentists from southern Canada fly in to provide services to the communities from time to time, on an often irregular cycle. Because of time constraints during these visits, not all individuals requiring care can be seen, and only the most serious cases are accommodated. As a result, residents who have an urgent need but are unable to obtain care may be flown from their communities to larger urban centres, at great expense, in order to receive necessary treatment. Children are frequently neglected in this service provider model, with only 63% of Inuit children aged six to fourteen years of age receiving oral health services in the previous year according to the *Inuit Oral Health Survey 2008–2009* [16].

### The policy stream: Dental therapists

A potentially effective alternative approach to ensuring adequate dental care in Inuit and First Nations communities has been to utilize dental therapists. Dental therapists are a mid-level oral health care provider described as a hybrid of a dental hygienist and a dentist. Dental therapists provide basic oral health care services such as fillings and extractions and preventive services at a lower cost than dentists and are trained as generalists who are well suited to provide care in rural and remote areas. Dental therapists are integral members of the oral health workforce in fifty-four countries, including developing countries such as Kiribati, Gambia, Mali, and Mozambique, as well as in highly developed, industrialized countries, such as the Netherlands, New Zealand, Australia, and Canada. Programs are offered by community colleges as well as universities depending on the jurisdiction. Dental therapy as a profession has a long history and the first dental therapy program began in 1921 in New Zealand. Programs typically vary in length from two to four years, with two years being the norm [21, 22].

Some research suggests that care delivered by dental therapists may decrease the total cost of oral health care, especially among children [22]. This finding may be attributed in part to the lower cost of training dental therapists as compared to dentists. In the United States, a twenty-eight month dental therapy program costs $65,500 USD per student, while a four-year dental school in the same state requires an investment of $160,000 USD per student. This significant difference in investment, both of time and money, allows dental therapists to be reimbursed at a lower rate than a dentist for providing similar care [23].

This reduction in cost does not appear to result in a sacrifice of quality in the services provided within the dental therapist’s scope of practice. Although they cannot perform all of the procedures that a dentist can, dental therapists do provide many high quality services [24–27]. A systematic review of twenty-three studies, spanning from 1950 to 2011, revealed that all but two studies concluded that dental therapists provide care at an acceptable level. All of these studies compared dental therapists to either dentists or dental students.

Nash and colleagues [23] found that dental therapists are highly accepted by people who use their services, however, a study conducted by Dyer and Robinson [28] in the United Kingdom, found different results. In a telephone survey of five hundred adults, only 15 % of participants knew that dental therapists existed, and only three people were able to identify correctly the duties they were permitted to perform. This indicates that, although the acceptance of dental therapists may be high in certain populations, a great deal of education may be necessary to allow clients and caregivers to give informed consent for treatment by a dental therapist [28]. It is reasonable to assume that a survey in Canada would yield similar results, as the majority of people living in Canada have never received treatment from a dental therapist.

### The politics stream: A brief history of dental therapy in Canada

Dental therapy in Canada has been heavily influenced by three main events. These are: (1) the federal government’s creation of the Ad Hoc Committee on Dental Auxiliaries; (2) the implementation and discontinuation of the Saskatchewan Children’s Oral Health Plan; and (3) the federal government’s funding and de-funding of two dental therapy training programs.

#### The ad hoc committee on dental auxiliaries

In June 1968, the Canadian Minister of National Health and Welfare announced that a committee on Dental Auxiliaries would be formed to “study all aspects of dental auxiliaries, especially as to the possible contribution auxiliaries could make in helping achieve better dental health for Canadians” [5, p. v]. Part of the reason for creating this committee was the growing body of evidence showing limited access to dental services among specific populations, and the resulting inequity in oral health care across communities. This committee was composed of both lay and professional members representing multiple health disciplines. Auxiliaries, as discussed in the report, were defined as members of a supportive oral health team, whose purpose was to assist the dentist in providing oral health care. The auxiliaries described in the report were dental assistants, dental hygienists, and dental technicians. The primary task of the committee was to examine the role of these existing dental auxiliaries and to investigate the addition of an expanded role dental therapist who would be a mid-level provider able to perform the tasks of a dental hygienist as well as some of the more routine tasks within the dentist’s scope of practice.

In 1970, the Ad Hoc Committee on Dental Auxiliaries Report was released. It was estimated that the existing oral health care workforce in Canada could provide only about one-third of the dental treatment services needed by the population. The report also stated that the higher education and income levels being achieved by many, rapid urbanization, and the growing number of insurance programs would likely lead to an even greater demand for dental services [5]. The members of the committee also believed, based on the report of the Hall Commission [29], that oral health care would be universally available by the 1980s in a fashion similar to medical care. At this time, there was interest in having a dental auxiliary who could be responsible for more standardized and routine tasks. The model of a dental nurse or dental therapist had already been adopted in several countries and the ad hoc committee on dental auxiliaries felt that this type of delegation would work well in Canada. There was also a third approach presented in the report, a type of dental therapist that existed in the Canadian Forces. These individuals were dental hygienists who, after obtaining three and one half years of experience, were eligible to take a fourteen week course which provided them with the necessary skills to perform additional duties. A similar model evolved in Canada’s Yukon territory, where the dental profession legislation was amended in 1964 to expand the scope of practice of the dental hygienists to the point that they resembled dental nurses/therapists from the United Kingdom.

#### The Saskatchewan children’s oral health plan

In 1972, shortly following the release of the Ad Hoc Committee on Dental Auxiliaries report, the Saskatchewan Department of Public Health introduced the Children’s Oral Health Plan. It was intended to address the unmet oral health needs of children from the ages of three to twelve. The oral care funded within this plan was to be performed primarily by dental therapists within elementary schools. The plan provided free coverage for school children and included an extensive range of services which were provided in school-based clinics, by dental therapists. The program was gradually expanded to include children between the ages of three and fifteen. [30, 31]. The decision to utilize dental therapists in this capacity did not sit well with the College of Dental Surgeons of Saskatchewan (which was also the Saskatchewan Dental Association at the time) and they released their own version of a dental plan for children in 1973, where private practice dentists were responsible for the delivery of the program. “The provincial dental association was highly critical of the provincial government’s planned delivery system and the introduction of unsupervised dental therapists to provide dental treatment for children” [[22], p. 247].

While dental therapists were originally trained and hired by the federal and provincial governments to provide care in public settings, in 1978, the *Dental Professions Act* allowed dental therapists, working under the supervision of a dentist, to work in private practice in the province of Saskatchewan [32]. This policy change has had a negative impact on the ability of the federal government to be able to recruit and retain adequate number of dental therapists to fill salaried positions in the territories. The change in legislation allowing dental therapists to work in private practice provided them with greater choices for employment. Dental therapists were now able to stay in urban centres rather than travelling or moving to remote communities; contrary to the original intent of the funding of dental therapy programs. This policy change may have been the beginning of the fall of dental therapy in that there were no longer as many dental therapists willing and available to work in remote areas with the vulnerable populations.

The voice of organized dentistry had been instrumental in raising concerns over the public health run plan and in making a case for an alternative, dentist focused model. Part of the original reason for the public health plan was the geographical disparities in access to oral health care services. As the University of Saskatchewan graduated more dentists, and these dentists wanted to attract more patients, their opposition to the dental therapist focused public health program grew. As a result, the combined provincial dental association/regulatory body aggressively lobbied the provincial government to discontinue the publically administered program that relied heavily on dental therapists. Eventually, the provincial government decided to privatize the program. The Saskatchewan provincial government, led by Premier Grant Devine, had been experiencing problems with fifteen of its cabinet ministers being convicted of fraud, four of whom served time in a correctional facility [33]. In 1993, the New Democratic Party came to power, and discontinued the program entirely [31]. The cancellation of the dental therapy program and selling of the equipment was not popular with the government opposition.

The Saskatchewan Dental Association was not the only group that was opposed to utilizing dental therapists. In 1980, the Canadian Dental Association, in a meeting that included representatives from the federal government, provincial dental associations, and native representatives, stated that dental therapists were practicing illegally in the provinces, as they had not been issued licenses to practice dentistry. The Canadian Dental Association made its position clear, noting that “all irreversible therapeutic dental acts or services must only be rendered by a licensed dentist” [[22], p.218]. When examining the list of participants in this discussion, the absence of dental therapists cannot be overlooked. The larger number of dentists, the strength of their organizations, their well-developed relationships with government, as well as their policy legacy of being the primary provider of dental services in Canada, severely overshadowed the small voice of dental public health specialists and dental therapists who were advocating for the dental therapy profession.

#### The federal government’s funding and de-funding of dental therapy training programs

In September of 1972, two new dental training programs began [30]. One was a program was originally for dental nurses (later the title was changed to dental therapists) run by the Wascana Institute of Applied Arts out of the Regina General Hospital in Saskatchewan. The second was the National School of Dental Therapy (NSDT) which was formed through a partnership between the Medical Services Branch for the Canadian federal government and the University of Toronto. It was first opened in Fort Smith, Northwest Territories but was moved to Prince Albert, Saskatchewan in 1981 [30]. The NSDT had been established through funding provided by Health Canada which allowed for tuition-free education for students enrolled in the program. Attempts were made to attract students from northern communities in the hope that they would return home upon graduation. It was a two-year program, available as direct entry for students from high school. In 1972, the only practice option for dental therapists was to be employees of the federal government in order to provide care to residents in remote or underserviced areas. The first graduates were hired by the federal government in 1974 to provide services in Yukon and the Northwest Territories [30]. A few other very small training programs in Saskatchewan and Manitoba followed.

Much of the classroom time in these programs was dedicated to practical hands-on training in the school’s dental clinic as well as in the field in rural communities. Dental therapists, during their twenty months of training, receive at least as much hands-on experience as graduates of a typical four-year dental school [34]. Upon graduation, dental therapists could perform routine oral health procedures including placing fillings, administering local anaesthetic, taking x-rays, removing teeth, placing stainless steel crowns, using anti-cavity agents such as fluoride, placing dental sealants, placing stitches, and performing recall examinations. They could not perform more complex procedures such as root canals, implants, and oral surgery. Generally, dental therapists work under the direction and indirect control of a dentist and complete dental services as outlined in a specific treatment plan that has been prepared by a dentist. A dental therapist may, however, provide an examination and oral diagnosis in an emergency, particularly when a dentist is unavailable. These emergencies could include complications arising from the removal of teeth, such as swelling or infection [35].

As dental therapists began working in urban centres, and the perceived need for dental therapist diminished, most programs closed. In 1993, the Saskatchewan Indian Federated College was awarded the contract to run the only remaining dental therapy program in Canada. In 2003, this school became known as First Nations University of Canada. Between 2003 and 2007, there were seventy-three graduates of the dental therapy program at the First Nations University of Canada’s National School of Dental Therapy (NSDT) [36]. A telephone survey of these graduates revealed that thirty-two of the graduates (62%) were working in private practice in the province of Saskatchewan, twenty were working in public health programs, and twelve individuals were not working [36]. In 2009, the federal government announced that funding for the National School of Dental Therapy at this school would be cut and the school closed down [30].

In 2009, the federal government undertook a review of the school, examining the content of the curriculum and the mandate of the program. Dental therapists were now working in private practice in Saskatchewan and Manitoba. After the review, the federal government made a public statement, declaring that the NSDT had transformed into an educational program, rather than a service providing program. Since the funding was provided on the premise that the school was providing an invaluable service, rather than mere education, the federal funding was eliminated in 2011 [37].

#### The current situation

The remaining dental therapists in the province of Saskatchewan are self-regulated and are required to register and be licensed with the Saskatchewan Dental Therapists Association [31]. In the 2013–2014 registration year, 238 individuals held a practicing license in the clinical restorative practice category; five held a public health preventive practice license; twenty-four were non-practicing members and twenty-three were affiliates. Forty-two members indicated they had qualifications as a dental hygienist. Dental therapists in Saskatchewan may work in private practice or as employees of Health Canada. It has been reported that, in 2013, more than half (144) of the Saskatchewan dental therapists were working in private practice [38]. In Manitoba, dental therapists must register with the dental association and may work in private practice or as employees of Health Canada. Dental therapists in British Columbia, where they are employees of the First Nations Health Authority, register with the dental association. Dental therapists working in all three territories need to be registered with the professional licensing department of the government. In Nova Scotia, Prince Edward Island, New Brunswick, Newfoundland, and Alberta, dental therapists may only work as employees of Health Canada. In Quebec and Ontario, dental therapists are not authorized to work since the activities they perform require them to be members of a regulatory college and the profession is not recognized in these provinces.

## Discussion

Several factors contributed to the federal government’s de-funding of dental therapy programs. The ability of dental therapists to obtain employment in the private sector for higher salaries contributed to the federal government’s difficulty in recruiting these individuals for public service in northern and rural communities. In the end, limited ability to recruit dental therapists for federal government positions, the longstanding resistance to the profession from organized dentistry, and the high costs of funding the education program with little perceived return on the investment overshadowed the benefits of continuing to fund dental therapy programs. This de-funding effectively put an end to the supply of new dental therapists in Canada.

At the time the funding of the final remaining dental therapy program (the NSDT program) was discontinued, students were graduating with a set of skills that enabled them to be reimbursed in private practice at a higher rate than if they chose to pursue a federal government position, where, in addition to the lower reimbursement rate, they would be working in a remote location, isolated from other dental professionals [36]. Since dental therapists had been granted the ability to work in private practice in some provinces, the federal government chose not to fund the education of providers who were remaining in urban centres rather than caring for vulnerable populations in remote areas. However, from a purely financial perspective, the funding of the NSDT seemed to be cost effective. Health Canada had reported that the money invested in providing a dental therapist’s education was recouped after each dental therapist had served two and one-half years in public service. However, since thirty-two graduates of the program between 2003 and 2007 decided to work in private practice rather than work in a publicly funded position, Health Canada lost the equivalent of eighty-eight years of service provision [36].

It was also difficult to overcome the persistent lobbying efforts of the dentistry profession. Dentistry has a rich institutional history, including professional associations, educational institutions, and government involvement in the regulation of the professions, and the provision of services through public health agencies. These institutions at various times have both enabled and hindered the evolution of dental therapy as a profession in Canada. In a 2010 study titled *The Saskatchewan Children’s Dental Plan: Is it time for renewal?* Ewart, is quoted as stating “… Dentists in private practice had the ear of the government and both parties had the same objectives, to let the market dictate the price of buying services” [22], p. 255]. The decision to move the dental program to the private sector, a switch which took place in 1987, has had a negative impact on the oral health of children in Saskatchewan. Reports released in 1999 prompted newspaper headlines stating that Saskatchewan children had third-world teeth [22]. Ewart, concluded by stating “it would be in the best interest of this province, and the people that reside within this province, to have the government assume a true leadership role and take the initiative to establish a dental program for children ‘because the state needs to be involved to implement what the marketplace cannot’” [[22], p. 256].

Although organized dentistry had embraced dental therapy into private practices in Saskatchewan and Manitoba, at least in a limited way, there was still strong resistance to the profession in other parts of Canada. The National Aboriginal Health Organization, in their 2003 discussion paper on dental therapy, stated “there is opposition from dentists’ associations, who see dental therapy as a threat to their profession. This is particularly true of dental associations in Ontario and Quebec, where dental therapists are not permitted to practise at all” [[39], p. 11]. The continued lack of familiarity with the profession of dental therapy outside of Saskatchewan and remote northern communities, as well as the small number of dental therapists remaining in publicly funded positions, made it difficult to attract the attention of the general public at the time of the school’s closing.

The Ad Hoc Committee on Dental Auxiliaries, formed in 1968, had twelve members, including both lay people and professionals. The composition of the committee demonstrates that the interests of organized dentistry were well represented. Only one member of the committee represented dental auxiliaries, while the Canadian Dental Association was able to nominate four dentists to represent private practice. It is also interesting to note that the individual chosen to represent dental auxiliaries was, in fact, a dentist [5]. This is far from surprising, especially given the recognized status of the profession of dentistry in comparison to the other oral health professions. The Canadian Dental Hygienist Association president wrote to the chairperson of the Ad Hoc committee requesting that dental hygienists be represented on the committee, rather than being represented by a dentist who spoke on behalf of all dental auxiliaries. Her request was acknowledged but not granted. The inability to obtain direct representation on the ad hoc committee and to have a voice at future decision-making tables may be one reason why the scope of practice of dental hygiene was not expanded to include the dental therapy skillset. In addition, private practice dentists were beginning to realize the economic advantages of having dental hygienists in their practice and may have been unwilling to lose them to government positions in remote communities.

While the Health Council of Canada argued in favor of supporting the dental therapist model [37], opposition to the decision to close the training institution was relatively weak and unorganized. Concern was expressed by Western Arctic NDP MP Dennis Bevington, who questioned Leona Aglukkaq, the national health minister, in the House of Commons on May 1, 2013. He wondered why the decision to close the training institution for dental therapists was made given that the oral health of his constituents was so poor. Minister Aglukkaq deflected the question by stating that the federal government invested in research and continued to increase health transfers to the provinces and territories, as the provincial governments were in a better position to know how to invest the money [40]. Unfortunately, Mr. Bevington did not have a strong lobby of concerned citizens voicing their concern. The weak political lobby of the Inuit and First Nations communities who were most directly affected by the closure meant that the backlash against the decision was not felt across the political landscape.

Inuit and First Nation population’s accessibility to health care in Canada is complicated by the fact that these communities are often small and scattered across Canada, frequently in rural or remote locations. This geographic issue is one of the factors limiting these subpopulations access to appropriate health care, as it is often difficult to recruit and retain health providers in remote locations [41]. The original dental therapy programs were designed to attract Inuit and First Nations students who would return to their communities but it is not clear how many students accepted into these programs were not Inuit or First Nations status. In addition, the unique cultural perspectives held by Inuit and First Nations people are often not well understood by non-Indigenous health care providers. These perspectives have evolved out of a history of federal government policies that limited the autonomy and power of Inuit and First Nations people. This is one reason why there may be benefits to having indigenous patients receiving services within their own communities [42].

Perhaps more importantly, health care in Canada falls predominantly under provincial jurisdiction, however, services for Inuit and First Nations people are largely the responsibility of the federal government and services are covered under its non-insured health benefits program. This blended responsibility for health care makes addressing equity issues less than straightforward as the division of federal and provincial powers creates a policy legacy that constrains future policy options. Policies designed to address equity issues in individual provinces have therefore evolved independently as windows of opportunity arose in various provinces leaving a patch-work of policies and access to care across the country.

A positive development has been the federal governments Children’s Oral Health Initiative. Pilot programs began in 2004 and have now expanded to include 320 of the 636 eligible Inuit and First Nations communities in Canada’s north [43]. Preventive oral health services are provided by dental therapists and dental hygienists in most provinces and just dental hygienists in Ontario and Quebec. While the program is innovative in creating community oral health workers to assist these health professionals, its long-term success will be impacted by the limited availability of dental therapists. In addition, the initiative is limited to communities that agree to make the program truly community-based leaving almost half of the Inuit and First Nations Communities without the program in place. Moreover, the program only provides preventive services and only to children under 8 years of age, pregnant women, and caregivers [43]. The program therefore does not ensure access to acute care and it remains unclear whether there has been an actual reduction in disease.

Will dental therapy rise from the ashes? Despite general support for policies that would enhance equity for dental care, overcoming the power and influence of the dominant interests to reinstate funding for a dental therapy program in Canada seems unlikely.

According to 2012 membership statistics of the Saskatchewan Dental Therapists Association, almost 60 % of the dental therapists in Saskatchewan, where the largest concentration of dental therapists reside, were forty years of age or older. One quarter of Saskatchewan’s dental therapists were reported to be fifty-five years of age or older [36]. Unless an alternative training program is introduced in the near future, it is very likely that dental therapy will cease to exist as a Canadian profession within the generation. Despite this, Canadian educational institutions possess substantial capacity to expand existing dental and dental hygiene programs to either include dental therapy training or expand the training of dental hygienists.

Given that the federal government has responsibility for Inuit and First Nations health and the provincial governments have responsibility more broadly for both health care and education, any country-wide policy change would require leadership from the federal government and cooperation from the provincial governments. Our analysis suggests that while a technically feasible policy solution is available (re-instating funding for dental therapy education programs) there continues to be no politically acceptable solution and thus Kingdon’s three streams will not align. As a result, it remains highly unlikely that a window of opportunity for policy change will open any time soon. So what other policy options exist? With the absence of federal government leadership, and the related possibility of a “big bang” policy change, the most viable option forward may be for incremental policy change at the provincial level. We have seen that such incremental change can be a practical alternative to more radical policy change [44].

The provinces have demonstrated an increasing willingness to support the expanded scope of practice of various professions as an approach to enhancing access to health care services as well as a means to controlling costs [45, 46]. Based on this experience, expanding the scope of practice for dental hygienists may be may a viable, if only incremental, policy option for enhancing access to case for Inuit and First Nations communities.

The global interest in the model of dual certification as a dental therapist and a dental hygienist makes incorporating dental therapy training into dental hygiene schools a viable option to consider. The research indicates that this model has great potential, not only to supply dental therapists to publicly funded positions, but also to increase the procedures that a dentist could perform in a private practice setting. The PEW research centre recently published an infographic on expanding the dental workforce which reported that 5157 additional procedures could be performed by a dentist practicing in a solo, private practice in one year by adding a dental hygienist provider with dental therapy training to the office staff [47].

Providing advanced designation and training to dental hygienists to provide expand their scope of practice could potentially prepare them well to work more independently in northern and remote communities, thus ensuring that Inuit and First Nations communities have access to at least basic services.

## Conclusion

Through a policy analysis of the rise and fall of the dental therapy profession in Canada we seek to advance our understanding of the policymaking process, past policy change, and the likelihood of future policy change that will enhance equity of access to dental health care for first nations and Inuit in Canada. The continued inability of many Inuit and First Nations Canadians to access appropriate oral health care providers in their home communities raises serious concerns about both equity of access and the health of these populations. Obtaining equity in access to health care services is generally thought of as a shared value within Canadian society. However, policy change is rarely achieved based on the strength of evidence or lobbying of government officials. It is essential that the complexities of the political process are fully taken into consideration if real change is to occur [6]. In this, and many other situations, the failure to implement effective policy solutions is complicated by the overlapping jurisdictions between the federal and provincial governments and the absence of leadership in coordinating an effective response. Whenever we see a policy problem that requires the coordinated attention of both the federal and provincial governments we can generally expect continued inadequate policy action.

## Abbreviations

CHMS: Canadian Health Measures Survey
FNOHS: First Nations Oral Health Survey
IOHS: Inuit Oral Health Survey
NIHB: Non-Insured Health Benefits Program
NSDT: National School of Dental Therapy
